# Emergency Department Overcrowding and Ambulance Turnaround Time

**DOI:** 10.1371/journal.pone.0130758

**Published:** 2015-06-26

**Authors:** Yu Jin Lee, Sang Do Shin, Eui Jung Lee, Jin Seong Cho, Won Chul Cha

**Affiliations:** 1 Department of Emergency Medicine, Seoul National University Hospital, Seoul, Korea; 2 Laboratory of Emergency Medical Services, Seoul National University Hospital Biomedical Research Institute, Seoul, Korea; 3 Department of Social and Preventive Medicine, Inha University Graduate School of Medicine, Seoul, Korea; 4 Department of Emergency Medicine, Gachon University Gil Hospital, Seoul, Korea; 5 Department of Emergency Medicine, Samsung Medical Center, Sungkyunkwan University School of Medicine, Seoul, Korea; Azienda Ospedaliero-Universitaria Careggi, ITALY

## Abstract

**Objective:**

The aims of this study were to describe overcrowding in regional emergency departments in Seoul, Korea and evaluate the effect of crowdedness on ambulance turnaround time.

**Methods:**

This study was conducted between January 2010 and December 2010. Patients who were transported by 119-responding ambulances to 28 emergency centers within Seoul were eligible for enrollment. Overcrowding was defined as the average occupancy rate, which was equal to the average number of patients staying in an emergency department (ED) for 4 hours divided by the number of beds in the ED. After selecting groups for final analysis, multi-level regression modeling (MLM) was performed with random-effects for EDs, to evaluate associations between occupancy rate and turnaround time.

**Results:**

Between January 2010 and December 2010, 163,659 patients transported to 28 EDs were enrolled. The median occupancy rate was 0.42 (range: 0.10-1.94; interquartile range (IQR): 0.20-0.76). Overcrowded EDs were more likely to have older patients, those with normal mentality, and non-trauma patients. Overcrowded EDs were more likely to have longer turnaround intervals and traveling distances. The MLM analysis showed that an increase of 1% in occupancy rate was associated with 0.02-minute decrease in turnaround interval (95% CI: 0.01 to 0.03). In subgroup analyses limited to EDs with occupancy rates over 100%, we also observed a 0.03 minute decrease in turnaround interval per 1% increase in occupancy rate (95% CI: 0.01 to 0.05).

**Conclusions:**

In this study, we found wide variation in emergency department crowding in a metropolitan Korean city. Our data indicate that ED overcrowding is negatively associated with turnaround interval with very small practical significance.

## Introduction

Emergency Department (ED) overcrowding has become a global health issue.[1–3] ED overcrowding stems from multiple input, throughput, and output factors.[4] It has been consistently reported that once overcrowded, an ED cannot function properly. Overcrowded EDs experience reduced performance quality as well as increased mortality and morbidity.[5, 6]

One of the most worrisome adverse effects of ED overcrowding is its effect on the prehospital system. Ambulances may be diverted from overcrowded EDs and rerouted to another facility, leading to additional transport time and increasing patient risks.[7–9] In addition, ambulances must wait at EDs until they find appropriate beds to unload patients from their stretchers, known as offload time. This prolongs the turnaround time and decreases the availability of ambulances.[9–13]

Most published studies have been conducted in developed Western communities. No studies focusing on the association between the prehospital system and ED overcrowding have been conducted in developing countries. Considering the differences in the emergency medical service (EMS) among nations, investigation of the effects of ED overcrowding in these countries is warranted.[14] The aim of this study was to describe the status of ED overcrowding in Seoul and evaluate the effect of overcrowding on ambulance turnaround time within this region.

## Materials and Methods

### Setting and participants

The study was conducted in Seoul, a large metropolitan city in Korea with a population of approximately 10 million people in an area of 605.2 km^2^. The population density of Seoul ranks among the highest for cities in the Organization for Economic Co-operation and Development (OECD).[15] There are 31 designated emergency centers in the region that are capable of treating major trauma and critical patients.

The EMS system in Seoul is controlled by the National Emergency Management Agency (NEMA) with call number 119. Ambulances responding to 119 calls cover all prehospital transport, free of charge, including basic life support (BLS), intravenous (IV) access, and endotracheal intubation. At the time of this study, there were approximately 112 ambulances responding to 119 calls in service. It is by law, that ambulance crews have to hand—over patients to hospital staffs directly, which implies that they must wait to unload the patient on appropriate beds.

Patients were enrolled if they were transported by ambulance to 28 emergency centers between January 2010 and December 2010. Patients transported to other hospitals were excluded, as we did not have data on overcrowding status for those institutes.

Ethics approval was obtained from the Institutional Review Board (IRB) of Samsung Medical Center to conduct this study: IRB No: 2014-07-058. The requirement to obtain written informed consent from all study participants was waived by the IRB. Patient records were anonymized and de-identified prior to analysis

### Methods and measurements

We used the National Emergency Department Information System (NEDIS) to calculate ED overcrowding status for each ED in Seoul. We defined overcrowding status as the average occupancy rate, which was calculated by dividing the average number of patients staying in an ED for 4 hours by the number of beds in the ED. This index has been shown to be a reliable tool to quantify overcrowding status.[16, 17]

The 119 ambulance run database was used to analyze ambulance turnaround time. This database is based on ambulance run sheets, which records patients’ clinical status along with time stamps for the time the 119 call was placed, engine start time, field arrival, field departure, ED arrival, and the time the ambulance returned to base. We defined the turnaround time as the interval from the time of ED arrival to the time the ambulance returned to base.

### Statistical analysis

First, we calculated the overall average overcrowding status for each ED. Then, we analyzed associations between overcrowding and turnaround time for each group. Regression analysis was performed to predict the turnaround time for each individual, with adjustment for potential confounders, using a multi-level regression model (MLM) with random effects for EDs.

We also performed a subgroup analysis using only the patients who visited EDs with occupancy rates over 100%. This analysis was done because the association between overcrowding and turnaround time may not be linear when occupancy is not saturated (i.e., 100% occupied). We used Stata software (version 12.1, Stata Corporation, College Station, TX) for all analyses.

### Sensitivity analysis

Emergency departments differ in level of overcrowding. We performed sensitivity analysis with same regression analysis methods on three subgroups. (average occupancy ≤0.5, occupancy 0.5 ~ 1.0, and occupancy ≥1.0)

## Results

**Fig 1:**
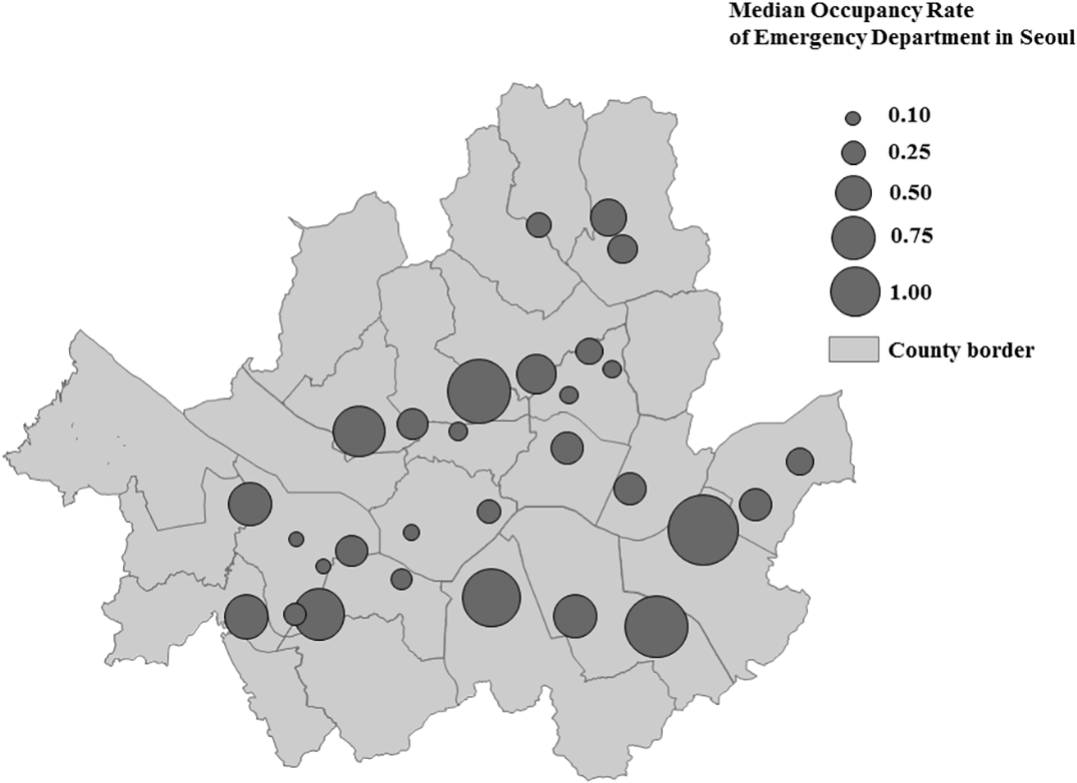
Median occupancy rate of emergency departments in Seoul.

From January 2010 to December 2010, 28 EDs in Seoul sent data to NEDIS. The occupancy rates of these hospitals are shown in Fig 1. The median occupancy rate was 0.42 (interquartile range (IQR): 0.20–0.76), with a minimum of 0.10 and maximum of 1.94. Because of the wide range, we stratified EDs into 4 groups according to their occupancy rate (Fig 1).

The 119 ambulance database from the same time period was used to enroll patients. During the study period, there were 277,214 ambulance transportations; 163,659 (59.0%) of these were transported to the 28 emergency centers evaluated in this study. The characteristics of the study population are shown in Table 1. The mean age was 49.7 years (standard deviation (SD): 23.1). Of the patients, 91,060 (55.6%) were male. 24,368 (14.9%) presented with abnormal mentality as verbal response to coma. 60,805 (37.2%) were trauma patients. The average turnaround time was 31.5 min (SD: 24.0). The average distance between the ED and base was 3.13 km (SD: 2.80).

**Table 1:** Characteristics of the study population.

	Mean occupancy rate				
Variables	≤0.5	>0.5 to ≤1.0	>1.0	Total	P
Number of EDs*	17	5	6	28	-
Number of patients	87,487	38,211	37,961	163,659	-
Age, mean (SD) **	49.1 (22.2)	49.0 (23.0)	51.6 (23.1)	49.7 (23.1)	<0.001
Male, N (%)	48,852 (55.8)	21,167 (55.4)	21,041 (55.4)	91,060 (55.4)	0.22
Abnormal mentality, N (%)	13,460 (15.4)	5,495 (14.4)	5,413 (14.3)	24,368 (14.9)	<0.001
Trauma, N (%)	36,460 (41.7)	14,015 (36.7)	10,330 (27.2)	60,805 (37.2)	<0.001
Turnaround interval, min (SD)	29.0 (22.3)	30.5 (22.9)	38.1 (27.1)	31.5 (24.0)	<0.001
ED-base distance, km (SD)	2.59 (2.10)	3.01 (2.30)	4.48 (3.97)	3.12 (2.80)	<0.001

EDs were grouped based on their mean occupancy rate. *

* ED: *emergency department*

**SD: *standard deviation*

**Fig 2:**
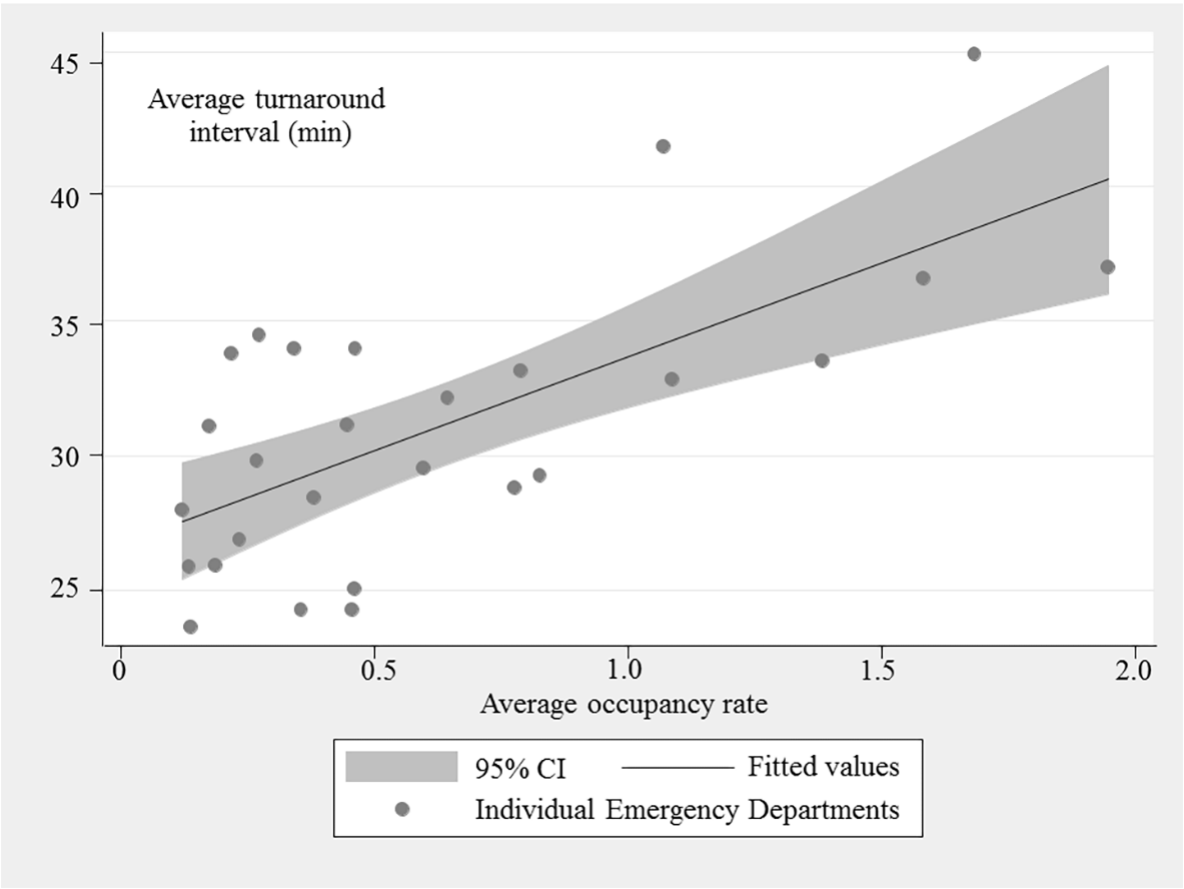
demonstrates the association between the average turnaround time and occupancy rate at the emergency department level. Spearman’s rho was 0.58 (P = 0.002). *95% CI: *95% confidence interval*

**Fig 3:**
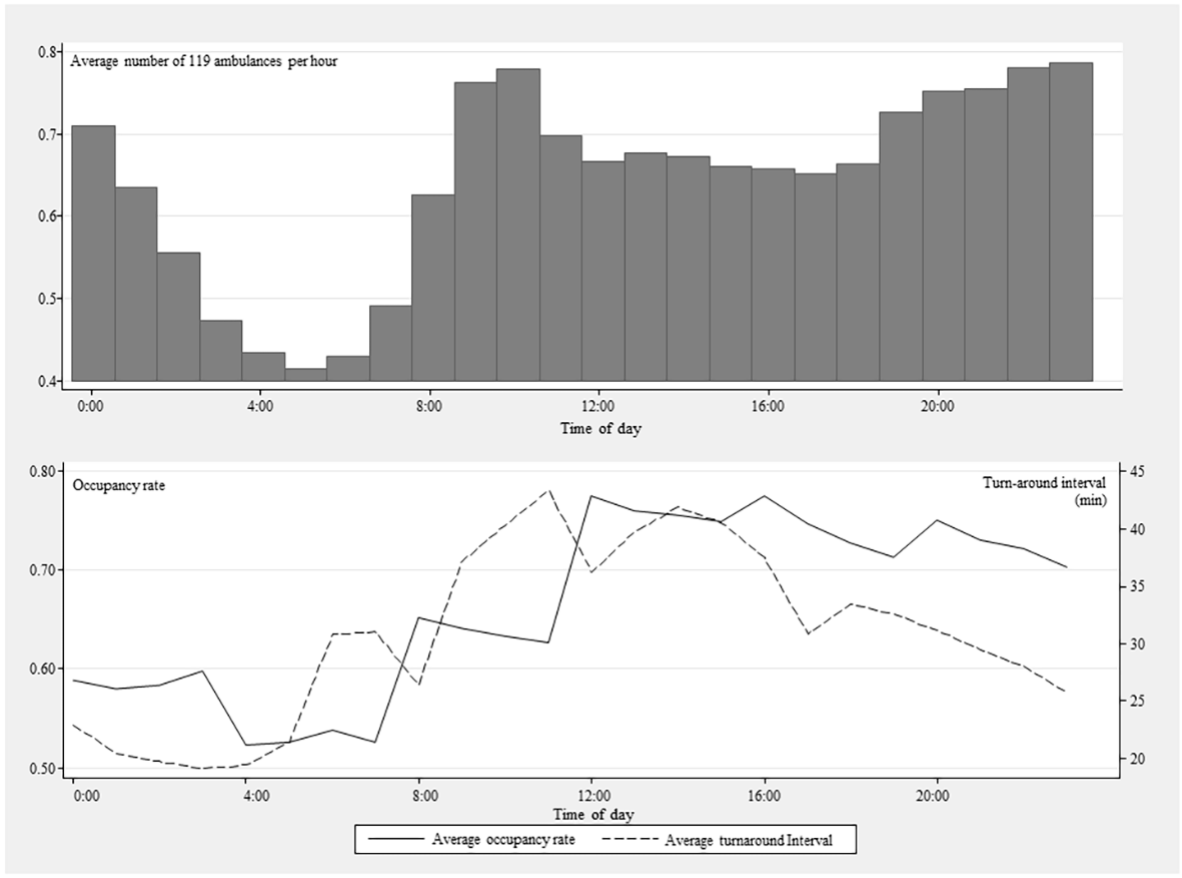
Circadian variation in emergency department overcrowding and ambulance turnaround time. During daytime, overcrowding and turnaround time increase together.

The association between occupancy rate and turnaround time for ED level is shown in Fig 2. Occupancy rate and turnaround time were positively associated (Spearman’s rho = 0.58; P = 0.002). However, the relationship within each ED differed widely. Traveling distance was also different among EDs. The within ED variation of turn-around time and distance was displayed in S1 Fig, and S2 Fig. In Fig 3, the circadian variation in turnaround time and occupancy rate is shown. Both turnaround time and occupancy rate increased during the daytime.

Table 2 shows the results of the linear regression analysis and multi-level regression model with random effects for EDs. An increase in 1% of occupancy rate was associated with 0.02 minute (95% CI: 0.01 to 0.03) decrease in turnaround time. When limited to patients treated at EDs with an occupancy rate of 100% or greater, we observed a 0.03 minute (95% CI: 0.01 to 0.05) decrease in turnaround time per 1% increase in occupancy rate (Table 3).

**Table 2:** Linear regression analysis with a multi-level regression model.

Factors	Multi-level analysis		
	Coef.	(95% CI) *	*P* value
**Age group, yr**			
**≥ 18**	0.00 (Reference)		
**19–65**	1.23	(0.88 to 1.59)	<0.001
**66–110**	1.89	(1.50 to 2.28)	<0.001
**Gender, male**	0.23	(0.02 to 0.44)	0.03
**Abnormal mentality**	3.26	(2.81 to 3.70)	<0.001
**Trauma**	1.41	(1.19 to 1.63)	<0.001
**Time**			
**00:00–04:00**	0.00 (Reference)		
**04:00–08:00**	4.70	(4.11 to 5.29)	<0.001
**08:00–12:00**	13.9	(13.3 to 14.4)	<0.001
**12:00–16:00**	15.9	(15.3 to 16.4)	<0.001
**16:00–20:00**	10.7	(10.1 to 11.2)	<0.001
**20:00–24:00**	6.69	(6.12 to 7.25)	<0.001
**Day of the week**			
**Sunday**	0.00 (Reference)		
**Monday**	2.23	(1.59 to 2.87)	<0.001
**Tuesday**	1.87	(1.23 to 2.51)	<0.001
**Wednesday**	1.54	(0.90 to 2.18)	<0.001
**Thursday**	2.05	(1.41 to 2.69)	<0.001
**Friday**	2.38	(1.75 to 30.2)	<0.001
**Saturday**	0.96	(0.30 to 1.62)	0.01
**Distance, km**	3.51	(3.44 to 3.60)	<0.001
**Occupancy rate, %**	-0.02	(-0.03 to -0.01)	0.001
**Constant**	12.2	(10.8 to 13.6)	<0.001

Covariates were distance between ED and ambulance base, age, gender, mentality, trauma, and overcrowding group.

*95% CI: *95% confidence interval*

**Table 3:** Subgroup analysis of EDs with occupancy rate > 1.0.

Factors	Multi-level analysis		
	Coef.	(95% CI) *	*P* value
**Age group, yr**			
** ≥ 18**	0.00 (Reference)		
** 19–65**	2.53	(1.76 to 3.31)	<0.001
** 66–110**	2.80	(1.97 to 3.63)	<0.001
**Gender, male**	0.62	(0.15 to 1.09)	0.01
**Abnormal mentality**	4.88	(4.21 to 5.56)	<0.001
**Trauma**	4.61	(1.08 to 2.15)	<0.001
**Time**			
** 00:00–04:00**	0.00 (Reference)		
** 04:00–08:00**	6.72	(2.34 to 11.1)	0.003
** 08:00–12:00**	12.7	(9.07 to 16.3)	<0.001
** 12:00–16:00**	15.1	(11.7 to 18.4)	<0.001
** 16:00–20:00**	13.4	(10.0 to 16.7)	<0.001
** 20:00–24:00**	8.49	(5.02 to 11.9)	<0.001
**Day of the week**			
** Sunday**	0.00 (Reference)		
** Monday**	3.75	(2.88 to 4.63)	<0.001
** Tuesday**	4.70	(3.79 to 5.61)	<0.001
** Wednesday**	4.82	(3.90 to 5.73)	<0.001
** Thursday**	3.78	(2.87 to 4.69)	<0.001
** Friday**	5.44	(4.53 to 6.35)	<0.001
** Saturday**	2.96	(2.07 to 3.85)	<0.001
**Distance, km**	2.88	(2.57 to 3.19)	<0.001
**Occupancy rate, %**	-0.03	(-0.05 to 0.01)	0.002
**Constant**	12.7	(8.95 to 16.4)	<0.001

Linear regression analysis with a multi-level regression model. Covariates were distance between ED and ambulance base, age, gender, mentality, trauma, and overcrowding group.

*95% CI: *95% confidence interval*

A sensitivity analysis was performed. Emergency departments with average occupancy rate below 0.5 showed positive association between occupancy rate and turnaround time without statistical significance. (Coef. = 0.03 (95% CI: -0.00 to 0.06, p = 0.08) Emergency departments with average occupancy rate between 0.5 to 1.0 showed positive association between occupancy rate and turnaround time without statistical significance. (Coef. = 0.03 (95% CI: -0.01 to 0.07, p = 0.10) Emergency departments with average occupancy rate above 1.0 showed positive association between occupancy rate and turnaround time without statistical significance. (Coef. = -0.05 (95% CI: -0.08 to -0.02, p<0.001)

## Discussion

ED overcrowding has become a major problem and is likely due to many causes, including increased complexity of clinical conditions, lack of hospital beds, and insufficient insurance programs. Overcrowded EDs function as gridlocks in the emergency health care system by diverting ambulances and increasing turnaround times. In this study, we described the overcrowding state among 28 major emergency centers in Seoul. However, the negative associations we observed between overcrowding and turnaround time were unexpected. Previous studies have demonstrated positive associations between ED overcrowding and turnaround time, while in this study, we observed a negative association. Similarly, subgroup analysis of EDs with occupancy rates greater than 1.0 also yielded a negative correlation

The discrepancy in outcome could be due to several different reasons. First, the cultures and policies of the EDs in this study may be different from those investigated in previous studies. ED staffs might have been more eager to find beds for 119 ambulance patients when the ED was overcrowded. The priority of getting vacant beds may be higher for 119 ambulances in the study settings though no national policy exists on priority of bed placement in ED. Though, the Fig 2 shows increasing turnaround time with occupancy rate which limits the potential influence of bed placement priority.

Second, ambulance patients could have been more likely to get up from stretchers in an overcrowded ED. Even though patients are transported by stretchers, it does not necessarily mean patients need beds for their stay in emergency departments. After the assessment by triage nurse and hand-over from 119 EMTs, many patients could sit up for chairs instead of beds. This may have facilitated the clearing process of 119 stretchers. However, we do not have data to show this process, which will be another limitation.

We observed a relative small effect size of a 0.02-minute decrease in turnaround time per 1% increase in occupancy rate, which is equivalent to a 2-minute decrease in turnaround time per 100% increase in occupancy rate. Although we had high statistical power due to the large sample size of this study, caution is needed in interpreting our findings.

ED overcrowding in developing countries has not been as widely discussed as in the developed countries. However, as the acute care system and EMS system of developing countries advance in size and performance, overcrowding may worsen. Since the healthcare system and basic characteristics of patients in these countries are distinct from those of the Western community, special attention is needed. Evidence-based policy and systematic evaluation of the “system disease” (overcrowding) will help us to mitigate this complex interwoven problem.[18]

### Limitation

This study has some major limitations. First, the definition of overcrowding was based on occupancy rate. Although the occupancy rate has been used as a surrogate marker for overcrowding in several studies and has been shown to be a reliable marker, there are other overcrowding indices, which may have altered our findings. For example, a substantial number of similar studies have used diversion status as a marker for overcrowding.

Second, the turnaround time could be biased by other factors. Though previous studies defined the turnaround interval as the period from ED arrival to the time the ambulance was back in service, this interval includes other processes, such as clearing and restocking ambulances, and completing paperwork.[7, 19–23] Carter et al. have evaluated the validity of using turnaround time as a proxy for offload time, and concluded that turnaround time can be readily used with caution.[20] The influence of traffic load is another critical confounder of the result. The decrease of turnaround time after office hour in the Fig 3 supports this relationship because the occupancy rate stays similar at the same time. However, we have adjusted for hour (with 4 hour interval) and day of week. Since the traffic has patterns of hours and days, the adjustment would have adjusted the potential confounding on some extent.

Third, the turnaround definition used in this article is different from previous studies, which defined turnaround time as the period from ED arrival to the time back in service.[22, 23] Although the meaning of back in service can be vague, in many circumstances, it means that the ambulance is leaving the ED. In our study, ambulances returned to base for clearing and restocking, increasing the turnaround time longer due to the added travel time. We attempted to correct this potential bias by adjusting for the distance from EDs to base.

Finally, there could have been other variables not included in the regression model that may have acted as confounders or major effect modifiers, which could explain our unexpected outcome.

## Conclusion

In this study, we found wide variation in emergency department crowding in a metropolitan city in a Southern Asian country. We also observed a significant negative association between ED crowding and ambulance turnaround time. However, the effect size was too small to have clinical and practical significance.

## Supporting Information

S1 Data The file S1_Data.csv is a file containing the data used in this study. (ZIP)

S1 Fig Association between overcrowding state (occupancy rate) and turn-around time is described. One can note a wide variation exit among emergency departments.
(TIF)

S2 Fig Association between overcrowding state (occupancy rate) and travel distance is described. Since the distance is associated with turn-around time and occupancy rate, it should be adjusted as a confounder of the regression model.
(TIF)
