# Supplementary figures and images for: Emergency Department Overcrowding and Ambulance Turnaround Time
Source: PLoS One. 2015 Jun 26;10(6):e0130758. doi: 10.1371/journal.pone.0130758 (PMC4482653; doi:10.1371/journal.pone.0130758)

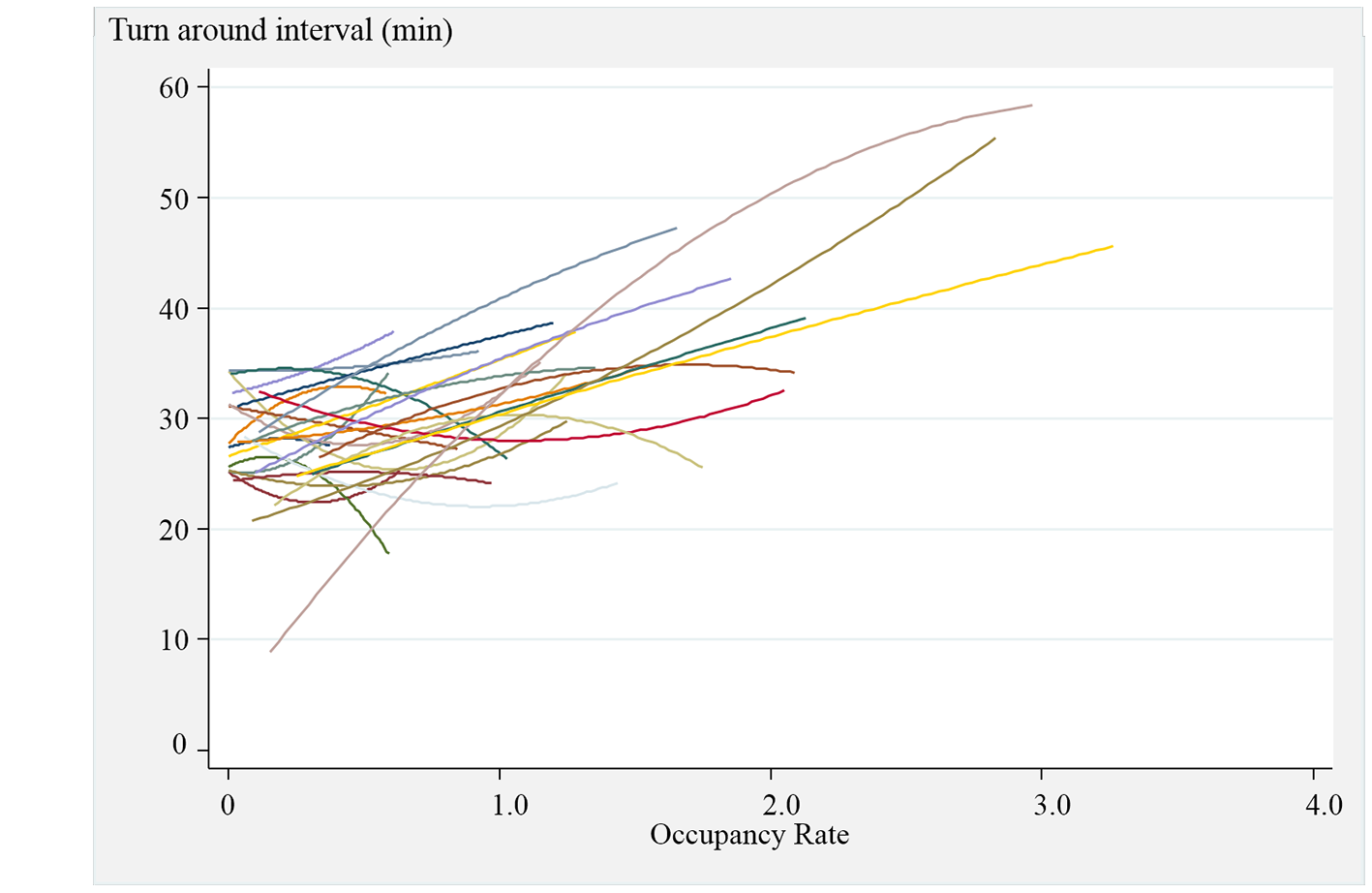

Supplement: S1 Fig — One can note a wide variation exit among emergency departments. (TIF) [file pone.0130758.s002.tif]

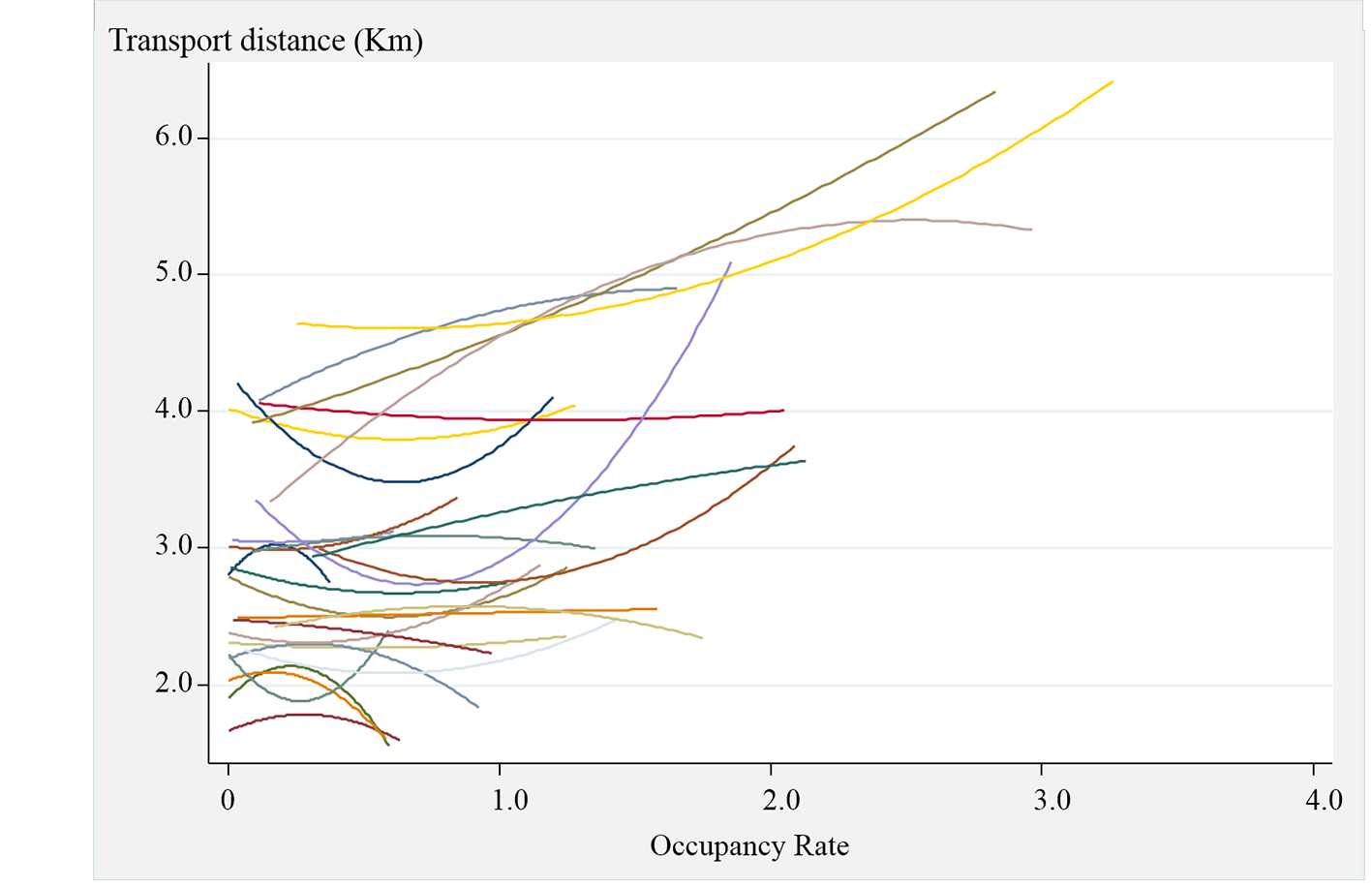

Supplement: S2 Fig — Since the distance is associated with turn-around time and occupancy rate, it should be adjusted as a confounder of the regression model. (TIF) [file pone.0130758.s003.tif]
